# Breaking Barriers with Humor and Heart: Dr. Elizabeth Hughes Fong’s Contributions to the Culturally Sensitive Practice of Applied Behavior Analysis

**DOI:** 10.1007/s40617-025-01083-8

**Published:** 2025-08-11

**Authors:** Robyn M. Catagnus, Lusineh Gharapetian, Adel Najdowski, Noor Syed, Jonathan Tarbox

**Affiliations:** 1 National University, Sanford College of Education – Applied Behavior Analysis, San Diego, CA USA; 2 Psychology, Pepperdine University, Los Angeles, CA USA; 3 Applied Behavior Analysis, Center for Autism Advocacy: Research, Education, and Supports, SUNY Empire State University, Saratoga Springs, NY USA; 4 Anderson Center International, Staatsburg, NY USA; 5 Psychology, University of Southern California, Los Angeles, CA USA

**Keywords:** Cultural sensitivity, Cultural humility, Diversity, Equity, Inclusion

With gratitude and grief, we dedicate this tribute to Lyz (known as Beth to her immediate family), an extraordinary person whose impact on all who knew her is immeasurable. Lyz passed away unexpectedly in early 2024. To her family, friends, colleagues, students, and those whose lives she impacted, she was a bright light that brought out the best in people through humor and kindness. Her story motivates us to continue her legacy of compassion, resilience, and belonging. This paper is a reflection on her life, a grief ritual, helping us cope by reading, writing, and discussing her memory to digest our profoundly painful loss (see Kestenbaum & Halsey, 2023). Even in death, Lyz united us; in this spirit, we come together to share the story of a pioneering figure in behavior analysis with gratitude for her friendship, and celebration of her life and extraordinary career. The behavior analytic community widely recognized Lyz as a leader in bringing diversity, equity, and inclusion (DEI) to the forefront of applied behavior analysis (ABA), advancing cultural sensitivity and diversity. Her career path included clinical roles, teaching and mentorship, research, editorial work, presentations, social media appearances, and policy-change efforts. Lyz understood the challenges faced by minorities and women in academic and business settings; this intersectional perspective fueled her advocacy for DEI within ABA.

Lyz’s deep commitment to DEI was apparent in the language she used to address cultural dynamics. To remain aligned with terms from her recent work, we have chosen to use “cultural sensitivity” throughout this paper from a variety of nuanced terms related to culture and practice (e.g., competence, agility, proficiency, humility, awareness, or effectiveness). That said, she varied terms based on the topic and focus of her work, and thus, we encourage readers to apply terminology for DEI based on context and situation.

Most quotations from Lyz in this paper come from the Behavior Speak podcast, specifically “Episode 53: Cultural Competence in Behaviour Analysis: How Far Have We Come? with Dr. Elizabeth Hughes-Fong, Ph.D., BCBA,” hosted by Ben Reiman (2022). We thank Mr. Reiman for the transcript of his podcast interview with Lyz. The quotes were transcribed with software, so punctuation and grammar errors may be present. For citations, we attribute the quotations to her, leaving them unedited to honor her words.

## Part I: Professional Journey

### Interdisciplinary Training

Lyz began her academic journey in psychology and counseling programs, wanting to become a school counselor. Her path to behavior analysis began after working and being mentored in an ABA setting, which led her to pursue certification in ABA. Her cross-disciplinary interests continued throughout her doctoral program in forensic psychology. A lifelong learner, she was interested in many different topics related to culturally sensitive services. For example, she explored racial disparities in health care, trauma-focused cognitive-behavioral therapies, and forensic and counseling approaches in addition to behavior analysis. It is possible that applying and integrating learning across fields later helped her innovate and lead more effectively (e.g., Kalbarczyk et al., 2018; Margolis et al., 2013).

During a guest appearance on the Behavior Speak Podcast with Ben Reiman, Lyz shared that she never expected to end up in ABA, but a mentor’s encouragement redirected her career (Hughes Fong, 2022). Adaptable and curious, she shifted her focus to ABA and transitioned away from her initial goal of becoming a school counselor. “I thought I was going to be a secondary school counselor. But then…I started the ABA coursework…and kind of never went back to counseling,” she reflected (Hughes Fong, 2022).

Lyz returned to clinical psychology after obtaining Board Certified Behavior Analyst® (BCBA®) certification, balancing full-time work and motherhood. Parent students are often nontraditional learners who may struggle to balance roles and responsibilities, reporting time-related, financial, health, and emotional challenges as they try to complete degree programs (Moreau & Kerner, 2015). Similarly, Lyz’s doctoral journey took two years longer than planned. She persisted, however, and earning a doctorate helped her transition to various roles in academia, where she made significant DEI contributions within the field of ABA.

### Professional Career and Leadership

Throughout her professional career, Lyz held roles across different fields, starting as a research assistant, school counseling intern, and behavior therapist, before advancing to a clinical leadership role in an ABA company. During her appearance on the Behavior Speak Podcast, she shared a childhood anecdote about enjoying a graphing assignment in school and mused, “…maybe I was always meant to be a behavior analyst” (Hughes Fong, 2022). Lyz quickly progressed to leadership roles in several behavior-analytic organizations, and started a private practice specializing in performance improvement and complex behavior cases with a strong emphasis and dedication to DEI.

Lyz recently stepped into academia, where she coordinated behavior analysis programs, taught courses at all levels, and supervised students toward certification. Lyz tailored her course content to enhance students’ cultural sensitivity by incorporating discussions of ethics, professional practice, single-subject research design, DEI, and social skills development, and she emphasized supporting diverse learners or those with special needs. Her scholarly research advocated for comprehensive guidelines and standards for effective intercultural practice. She was ahead of the times in doing so, as the field eventually shifted to supporting recommendations to apply these very same strategies to ABA coursework, graduate programming, and clinical practice (e.g., Hilton et al., 2021; Najdowski et al., 2021). Lyz was also a valued mentor to students and early-career professionals. Later in her career, she took on roles overseeing training programs and served as an expert consultant. Her students recall Lyz as funny, kind, and helpful.

In addition to teaching and research, Lyz was involved in community service and university committees, and she regularly participated in local events, such as career days and educational initiatives. She also co-created student programs, volunteered, and participated in student events.

Lyz engaged in extensive international service and dissemination, teaching culturally responsive practices in international settings such as Japan and South Africa. Japanese behavior analysts approached Lyz when she was the President of ABAI's Multicultural Association of Behavior Analysis (MultiABA) special interest group (SIG). They requested her help to establish BCBA and BCaBA® course sequences in Japan. Despite a rich history and many local ABA experts, they had not yet established a certification system. As a result, BCBAs were scarce in Japan. Lyz recognized the cultural disparities and facilitated the development of the verified course sequence (VCS) in Japan. Once the training program officially began, Lyz continued supporting it (K. Matsuda, personal communication, July 30, 2024). Whether organizing local community events, teaching, or disseminating ABA globally, Lyz always tried to improve the world.

### Scholarship and Dissemination

“I also tend to look outside of ABA, to fields such as psychology, who have done a superior job addressing the need to examine the role that culture plays in treatment” (Hughes Fong, Curriculum Vitae). Lyz began writing, publishing, and presenting peer-reviewed research early in her career. Her publications and national and international presentations centered on applying behavior analysis to multicultural populations, telehealth, and social validity. Over nearly 15 years, her public presentation topics and journal articles increasingly focused on DEI within ABA. Her cross-disciplinary approach allowed her to incorporate best practices from related fields to advocate effectively for DEI within ABA.

Importantly, Lyz discussed culture and recommendations for DEI through a behavioral lens. For example, her works frequently referenced various books and articles by B.F. Skinner on the topic of culture, described cultural values as reinforcers, and framed recommendations for cultural sensitivity through the lens of cultural and organizational contingencies (e.g., Fong et al., 2016; Fong & Lee, 2017; Fong et al., 2017; Fong, 2020a, 2020b; Hughes Fong, 2021a).

Interestingly, Lyz did not particularly enjoy writing. Speaking engagements made her anxious *every time*, yet she still gave many influential talks and presentations during her career. Her presentations focused on culturally relevant topics and the need for inclusivity and diversity in ABA. She thought deeply about how to improve behavior analysts’ ability to address cultural and demographic variables.

### Publications

Lyz’s published works (see Table 1) were among the earliest in ABA to shine a light on the need to address culture and focused on the critical importance of cultural sensitivity or competence, ethical practice, and ongoing training related to culture. Her earliest publication as it relates to such topics occurred in 2013, when in a break from the standard approach in behavior analysis at the time, Hughes Fong and Tanaka noted, “behavior analysts must be reminded that their professional practices and respectful disciplines, too, are never culturally neutral” (p.18). While behavior analysis is grounded in objective practices and scientific principles, it is nonetheless shaped by the cultural context in which it is applied. Lyz believed behavior analytic organizations must guide clinicians in culturally competent practice, and to that end, Fong and Tanaka (2013) proposed their seven standards for cultural competence in behavior analysis. These standards were developed after reviewing *The National Standards on Culturally and Linguistically Appropriate Services* (CLAS). Lyz often made the case that we should look to related human service fields that have made significant strides in advancing DEI initiatives, which may allow us to create strong, evidence-based frameworks to support and enhance DEI efforts within ABA. This would ultimately yield a more informed and strategic approach to fostering inclusivity and equity.

**Table 1:** List of Dr. Elizabeth Hughes Fong’s peer reviewed publications and abridged abstracts

Reference	Abstract
Hughes Fong, E. (2021a). Building a culturally and diversity-sensitive workforce. In J. K. Luiselli, R. M. Gardner, F. L. Bird, & H. Maguire (Eds.), *Organizational behavior management approaches for intellectual and developmental disabilities* (pp. 251–266). Routledge	This chapter examines diversity and its scope within the workplace. The importance of cultural competence is discussed through an analysis of historical workforce diversity practices. Several challenges to building a diverse workplace are identified and discussed, which include recruitment, eliminating microaggressions, and broadening diversity beyond race, ethnicity, and religion. If organizations can overcome these challenges, they are likely to build a culturally diverse workplace by using ongoing diversity assessments, setting meaningful goals, and developing effective trainings
Fong, E. H. (2020b). Standards for culturally sensitive practice of applied behavior analysis. In Conners & S. Capella (Eds.), *Multiculturalism and diversity in applied behavior analysis: Bridging theory and application.* (pp. 19–27). Routledge	This chapter will explore the concept of standards of culturally sensitive practice in the field of applied behavior analysis (ABA) and the issues related to cultural competence including its relevant ethics, challenges, and strengths
Fong, E. H. (2020a). Examining cross-cultural supervision in applied behavior analysis. In B. Conners & S. Capella (Eds.), *Multiculturalism and diversity in applied behavior analysis: Bridging theory and application.* (pp. 181–193). Routledge	This chapter will explore issues related to cross-cultural supervision, including its relevant ethics, challenges, and strengths. It is important for supervisor and supervisees to explore the potential impact of these factors and to address how to uphold clinically sound and ethical supervision experience
Fong, E. H., Ficklin, S., & Lee, H. Y. (2017). Increasing cultural understanding and diversity in applied behavior analysis. *Behavior Analysis: Research and Practice, 17*(2), 103–113	In recent years, the demands for behavior analysis to serve consumers with diverse cultural backgrounds have significantly increased. The field is in great need of culturally competent behavior analysts who can integrate appropriate cultural considerations to their programs. The field of behavior analysis can address this growing need by fostering cultural competency in professional training through increasing relevant training opportunities and the development of culture- and diversity-relevant educational curricula and materials, and by supporting efforts to increase the number of ethnically and racially diverse behavior-analytic workforces in academic and professional settings
Fong, E. H., & Lee, H. (2017). Sociocultural perspective on autism intervention. In M. Fitzgerald & J. Yip (Eds.), *Autism: Paradigms, recent research and clinical applications* (pp. 291–300)*.* IntechOpen	One approach to autism intervention is applied behavior analysis (ABA), which seeks to intervene on socially significant behavior. In addition, to using an approach such as ABA, which emphasize social significance, individuals may also use a cultural broker. The cultural broker can help to bridge the gap between parties and promote more effective treatment experience and thus help to ensure a more culturally sensitive approach to intervention
Fong, E. H., Catagnus, R. M., Brodhead, M. T., Quigley, S., & Field, S. (2016). Developing the cultural awareness skills of behavior analysts. *Behavior Analysis in Practice*, *9*, 84–94	The purpose of this paper is to provide suggestions to serve as a starting point for developing behavior analysts’ cultural awareness skills. We present strategies for understanding behavior analysts’ personal cultural values and contingencies and those of their clients, integrating cultural awareness practices into service delivery, supervision, and professional development, and becoming culturally aware in everyday practice
Fong, E. H. & Tanaka, S. (2013). Multicultural alliance of behavior analysis standards for cultural competence in behavior analysis. *The International Journal of Behavioral Consultation and Therapy*, *8*(2), 17–19	Due to the increase in culturally diverse populations working within the framework of behavior analysis, clinicians must ensure that they are properly educated and aware of cultural competences. This article will attempt to define culture, competence and cultural competence, as well as provide recommendations for application and future challenges

Soon after, her publications focused on the importance of developing cultural self-awareness skills (Fong et al., 2016; Fong & Lee, 2017; Fong et al., 2017). Identifying (or establishing) diversity as a value, maintaining respect for different cultures, traditions, and communication styles, and an awareness of how one’s own cultural beliefs and experiences shape identity and interactions is crucial for developing cultural sensitivity (Fong et al., 2016). Lyz described how racial microaggressions create significant barriers to diversity by causing mental exhaustion and stress, particularly for individuals of color in negative racial climates, ultimately hindering their ability to fully engage in professional and academic settings. These articles offered recommendations compiled from the literature to aid in building culturally sensitive professionals, including developing culturally relevant training, providing mentorship opportunities, supporting minority students and faculty, and creating a diversity-friendly campus environment.

While she initially focused on the overall importance of cultural sensitivity, she later called for specific intercultural standards and guidelines (Fong, 2020b), explored cross-cultural supervision (Fong, 2020a), and systemic changes to support DEI in ABA. In a book chapter exploring issues related to cross-cultural supervision (Fong, 2020a), she analyzed components of the BACB’s Professional and Ethical Compliance Code for Behavior Analysts (Behavior Analyst Certification Board, 2014) and the Fifth Edition Task List (Behavior Analyst Certification Board, 2017) that applied specifically to staff supervision tailored toward cultural sensitivity. For example, she suggested that supervisors could create defined goals around culturally sensitive service delivery, include cultural trainings or competencies in supervision, be sensitive to the language and terms used by a supervisee’s culture when providing feedback, and create a task analysis of what culturally sensitive supervision and practice might look like as a tool for both self-reflection and as a supervisee assessment.

More recently, Lyz shifted her focus toward establishing equitable access to academic conferences and addressing LGBTQIA + issues in the field. In her final years, she concentrated her efforts on systemic change and DEI initiatives, including publishing a chapter on establishing culturally sensitive workforces in organizational behavior management (Hughes Fong, 2021a). Specific to organizational health, she noted that an organizational culture that perpetuates racism harms employee well-being, stifles the growth of marginalized individuals, limits access to resources, and creates conflict between minority and majority groups (Hughes Fong, 2021a, b). Furthermore, in this chapter, Lyz underscored that diversity training alone was insufficient for translating knowledge, skills, and attitudes into practice. She emphasized the importance of maintaining a clear link between diversity training, the organization’s mission, and its culture. For meaningful behavioral change to occur, organizational contingencies must actively support these shifts (Hughes Fong, 2021a).

### Influence and Impact

Lyz established and edited journal sections dedicated to diversity in behavior analysis to influence the discourse around DEI. For example, as an associate editor for *Behavior Analysis: Research and Practice*, she initiated the “Diversity in Behavior Analysis” section launched in 2019. Over time, her editorial roles expanded, allowing her to shape the direction of research and practice in behavior analysis and ensure that issues of DEI were prominently featured.

Lyz’s efforts extended to actions for policy change. She called for culturally sensitive standards in behavior analysis across several forums (e.g., journal articles, conference presentations), and she generated interest in the importance of DEI through years of sustained advocacy. She wrote about integrating multicultural perspectives into ABA, then avidly volunteered for boards and committees to help shape policy and facilitate the systemic changes she recommended.

As an early advocate for DEI in ABA, her work evolved from participating in task forces and committees focused on DEI to founding SIGs dedicated to promoting cultural sensitivity and inclusivity. For example, early in her career, she founded MultiABA within ABAI in 2009. Later, she rebranded the group as the Culture and Diversity SIG. Lyz also served on several influential committees, including the Executive Committee for the American Psychological Association's Division 35. She strongly advocated for ABA to establish cultural competency standards and joined ABAI’s DEI Board. Over time, she collaborated on guidelines and policy statements for ABA professional organizations and higher education training programs. Her efforts eventually garnered significant interest, as evidenced by the frequent citations of her work by other authors. For example, according to Google Scholar, other authors cited “Developing the Cultural Awareness Skills of Behavior Analysts” (Fong et al., 2016) 339 times, “Increasing Cultural Understanding in Applied Behavior Analysis” (Fong et al., 2017) 161 times, and “Multicultural Alliance of Behavior Analysis Standards for Cultural Competence in Behavior Analysis” (Fong & Tanaka, 2013) 116 times.

Given that Lyz was an early contributor to DEI topics within behavior analytic literature, it is plausible that her scholarly work and dissemination efforts informed some of the changes to the BCBA Test Content Outline (6th edition; Behavior Analyst Certification Board, 2022) centered around DEI within behavior analysis. As the behavior analytic profession became more attuned to the needs of diverse populations, it began to incorporate more content related to cultural sensitivity and the importance of considering cultural variables in practice (Behavior Analyst Certification Board, 2017). The BACB’s Sixth Edition Test Content Outline (Behavior Analyst Certification Board, 2022) contains several items specifically focused on the cultural contexts of both practitioners and clients.

### Unfinished Work

Despite Lyz’s extensive efforts and contributions, her efforts aimed at establishing comprehensive guidelines for cultural sensitivity and diversity in behavior analysis remains unfinished. Neither the Association for Professional Behavior Analysts (APBA) nor ABAI have developed cultural and diversity guidelines for the practice of applied behavior analysis (Hughes Fong, 2022). She called for ABA organizations to create multicultural guidelines and standards, citing a lack of guiding frameworks as contributing to a critical gap in training programs. She identified challenges to progress, including the absence of a task force or committee to address multiculturalism in behavior analysis; a lack of consensus for establishing guidelines for culturally sensitive practice; and the frequent failure of researchers to report social validity and demographics in behavioral research publications (Fong, 2020b). Nevertheless, Lyz remained optimistic about the future of our field, identifying notable strengths, including the formation of an ABAI multiculturalism task force; components of our Ethics Code that are sound starting points for the development of such standards; and various steps being taken across conferences, within journals, and in webinars that highlight our field’s deficits and advocate for change (Fong, 2020b).

## Part II: Formative DEI Experiences

### Personal Background

Being an international adoptee and working with diverse clients helped to shape her professional perspective. “I’m adopted. So, I think that had a significant influence on my interests. I also have done most of my clinical work in urban areas working with low SES populations,” she shared (Hughes Fong, 2022). Her firsthand experiences exposed her to DEI gaps in ABA literature, particularly the lack of diverse communities reflected in the literature. She recalled, “The articles that were out there… didn’t really fit the population that I was working with” (Hughes Fong, 2022).

Despite initially feeling alienated within the ABA community due to her nonbehavioral educational background, Lyz’s resolve only strengthened. “I remember when I started in the field feeling like I didn’t fit in because my master’s degree wasn’t in behavior analysis and I didn’t study in a lab or anything like that,” she admitted (Hughes Fong, 2022). Perhaps feeling like an outsider within her own ABA field gave her another personal experience of being in a marginalized group. In addition to being an Asian American who did not feel “behavioral enough,” she also struggled with balancing work as a scholar and a professional, with protecting enough time in her life for her family. She knew firsthand how difficult it can be to balance professional and family life, especially when working remotely.

In personal conversations, she often talked about the overwhelm of mental labor that disproportionately affects mothers and female academics in the United States (Dean et al., 2021). Research confirms that Black Indigenous Persons of Color (BIPOC) women in academia often shoulder a heavier burden of emotional labor involving teaching, research, advising, and catering to the emotional needs of students, a service that is unrecognized and undervalued (Choi, 2023). This imbalance adversely affects female faculty of color, limiting their professional advancement (Misra et al., 2021). Though female faculty in the US typically perform more service than their male counterparts, they also have a lower institutional rank (Guarino & Borden, 2017; Johnson, 2017). Such inequities suggest a lack of support or appreciation for women’s academic contributions.

### Experiences of Exclusion

Like many BIPOC professionals, Lyz experienced stereotyping, microaggressions, and discrimination related to her gender or race (Endo, 2015; Keum et al., 2018; Li, 2014). In a keynote presentation, she shared a story about male colleagues condescendingly telling her to “put on her big girl pants” (Hughes Fong, 2021b). Additionally, she encountered insensitive remarks about her appearance, such as being asked, “Are you ESL?” Marginalization extends beyond gender and race to varied intersecting identities, such as ethnicity, SES, or sexual orientation (Hughes Fong & Rosado, 2024). BIPOC women often encounter such racism or sexism in professional settings.

To friends, Lyz shared that her professional interactions with male colleagues occasionally included unsolicited attention, social media overtures, or socially inappropriate requests (e.g., surrogacy for a professional male colleague). Female professionals, conversely, sometimes treated her with disregard, a form of microaggression that renders a person “invisible” (Sue et al., 2007). Such adverse experiences exemplify the everyday realities of harassment and objectification that can impact the well-being of marginalized women (Buchanan et al., 2018). Despite or because of these experiences, she was very motivated to advocate for change (for a discussion of BIPOC women using adversity as motivation, see Chance, 2022).

### Impact of the Pandemic and Media

The COVID-19 pandemic increased attention to racial justice, and Lyz frequently spoke out against racist comments online, often discussing the systemic inequalities impacting marginalized communities. As xenophobia increased during the pandemic, so did her fear; she felt unwelcome in her community and worried about her family’s safety (e.g., Findling et al., 2022). Owing to both direct and vicarious racism, depression and fear were common among Asian and Black Americans during the pandemic (Chae et al., 2021). While much of Lyz’s works were geared toward advocating for cultural sensitivity in behavior analytic service delivery settings, her message is resoundingly universal in its applications to one’s personal and professional approach and general interactions with others. Her work noted that a critical element in combating racial injustice and building cultural sensitivity involved one’s personal and professional values.

George Floyd’s murder in 2020 sparked considerable dialogue in the ABA community about racism and police brutality. Lyz was concerned that too few of her colleagues took action—they posted online and talked but did not engage in change efforts (i.e., performative allyship; Sylvain et al., 2022). This gap between rhetoric and action for change, along with fear and overwork, resulted in a growing exhaustion. Her efforts toward social justice led to compassion and DEI fatigue—a common experience among BIPOC advocates (Doan & Kennedy, 2022).

Recently, Lyz expressed the frustration noted by many individuals from historically underrepresented communities: “It feels exhausting to me … Like how many more times do minority experiences have to be questioned? Especially because of a lack of data. The “data” are everywhere. Look at the news, who knows, even at your own neighborhoods, places of work. I want to know that if I experience racism, sexism, name your -ism that it’s not going to be written off. And it makes me sad that my colleagues are diminishing the lived experiences of their peers” (personal communication, February 16, 2023). She was experiencing the emotional and mental toll of DEI work.

In public talks, Lyz said the continuous resistance to inclusion initiatives felt personally painful. It is common for advocates and allies to experience DEI fatigue, but it is not easy to overcome (Doan & Kennedy, 2022). To cope, Lyz tried to make time for self-care, like spending time with family, working out, and running. She cultivated and maintained a network of close friendships, remaining upbeat most of the time.

In early 2024, Lyz told colleagues she felt physically ill sometimes, was “burned out,” and physically exhausted. She faced significant stress balancing her work and family responsibilities and, unbeknownst to her, was at risk of a severe health condition. Like many working mothers, Lyz prioritized day-to-day demands over her well-being, delaying or skipping medical appointments and annual screenings (Shin, 2019). This pattern, though common, can lead to health implications over time, including increased emotional exhaustion as well as physical illness. Avoiding regular health screenings and doctor exams can heighten worry, reduce well-being, and hinder the ability to manage dual roles. Linked to problems such as anxiety and depression, missed medical care may also lead to an increased risk of mortality (e.g., Charton et al., 2024; Hogg, 2023).

### Untimely, Tragic Loss

Lyz did not receive a diagnosis or treatment in time. Perhaps she would be with us today in a “different world,” with less overload and more societal support​​. Perhaps the adoption of workplace policies designed to support the well-being of employees, such as shorter workweeks, aligning work with children’s school schedules, or extended and paid family leave, could have helped her prioritize her health despite work and advocacy (Hogg, 2023).

Lyz’s family strongly encouraged her to see a doctor and take good care of herself. Unfortunately, managing health and doctor visits takes time and effort. It is too easy to allocate responses to other pressing responsibilities. We ask readers to reflect: *Are there aspects of health or well-being you need to prioritize? What steps could you take this week to be happier or healthier, even within the competing demands of life?*

## Part III: Strengths of Character

As we pivot from the adversities Lyz faced—ranging from burnout to systemic discrimination—we spotlight three of her essential qualities (i.e., behavioral repertoires) that we think sustained her pursuit of social justice. Lyz’s compassion and humor helped her navigate hardships with authenticity, even joy. This section discusses her leadership style and explains how she modeled resilience.

### Silence and Humor in the Context of Humble Leadership

#### “Expertless of the experts” (E.H. Fong, personal communication, April 1, 2021)

Lyz was a humble leader who was quiet in meetings and at events. Valuing others’ contributions fosters openness to new ideas, promotes psychological safety and reduces conflicts, and may enhance creativity (Black & Wiederhold, 2014; Wang et al., 2018). Silence can serve many functions. It can be conflict avoidance, listening, resistance to oppressive power dynamics, or a way to maintain control in difficult situations (Chen et al., 2019; Hershcovis et al., 2017). She often spoke of her efforts to take others’ perspectives, better understand people, build stronger relationships, and encourage diversity of thought (e.g., Catagnus & Rock, 2020).

Humor was also her coping mechanism for the discrimination and microaggressions she experienced in her professional endeavors. Lighthearted or dark, joking helped her deflect negativity and stay resilient. It was a way to slip from social visibility to withdrawal or introspection as needed (Borgella et al., 2020; Houshmand & Spanierman, 2021; Saucier et al., 2016). Both humor and silence helped her navigate complex topics or situations effectively and gracefully.

### Authentic, Inclusive Leadership

Our BIPOC clinicians and BIPOC communities have experienced years of neglect and trauma. “We, as behavior analysts, have failed them by failing to understand and take into account their diverse experiences and how these experiences contribute to who they are as a person…what their preferences are, etc. We have the ability to apply our science to a multitude of issues, so let’s do that” (E. Hughes Fong, personal communication, September 25, 2021).

As Lyz pointed out, many fields, including behavior analysis, need help to increase professional diversity. Students of ABA are increasingly diverse, but there is a discrepancy in their representation in leadership or advanced roles. For example, 16.71% of Registered Behavior Technicians (RBTs) are Black, whereas only 5.06% of BCBAs are Black (Behavior Analyst Certification Board, 2024). This discrepancy may also stem from a failure to align traditional views of professionalism with the identities of women and under-represented populations within a discipline (Rosenberg et al., 2021). Likewise, professionalism in ABA is often defined by established majority norms regarding how we dress, talk, or behave. Typical standards may unintentionally reinforce biases or discourage cultural expressions. In other words, current constructs of “professionalism” may disadvantage marginalized groups. It is possible to welcome a broader spectrum of workplace behaviors within a genuinely inclusive workplace (Mousseau, 2024).

By prioritizing meaningful contributions over superficial conformity, Lyz challenged some of the biases and barriers inherent in traditional professionalism (Minkin, 2023; World Economic Forum, 2024). Her relaxed approach helped others feel safe to share diverse perspectives at work or school. Balancing diversity, authenticity, and notions of professionalism can help advance DEI (see Brimmer, 2024, and Duran, 2022, for further discussion on how race intersects with perceptions of authenticity).

## Quiet Persistence

Early in her career, Lyz doubted the ABA community would welcome and support DEI efforts. Her initial projects and talks often faced lukewarm interest, evidenced by presentations in nearly empty rooms during conferences. A lack of initial engagement, however, did not deter her. She kept working toward her goals—often in the background, without branding or a social media presence. Her determination was evident again in 2013 when professional organizations did not adopt her proposed, co-authored guidelines for cultural competence in behavior analysis (Fong & Tanaka, 2013). She recalls the response as, effectively, “Thanks, but no thanks” (Hughes Fong, 2022). Despite setbacks or slow progress, she persisted. At times, she hoped for more acknowledgment of her professional value.

I had applied several times to be a full member of [an ABA professional organization] and I was always denied until just last year. Last year was… the first time they accepted me as a full member. … I wonder what changed for now all of a sudden. Because… I hadn’t published anything in a couple of years. So, what’s changed now that I’m worthy enough? I’m hoping that the reason that now they decide to make me a full member is because they’re seeing how important and relevant the issues are (Hughes Fong, 2022).

Wanting recognition but feeling unaccepted is a common experience for BIPOC female academics. Exacerbated by systemic issues such as racism and tokenism, these institutional barriers contribute to feelings of intellectual inferiority and marginalization within predominantly white institutions and often lead to imposter syndrome, constantly fearing exposure as a fraud despite evident success (Edwards, 2019). Further intensified by the lack of representation and role models, imposter syndrome disproportionately affects BIPOC women and those from the LGBTQIA + community, hindering their professional growth and sense of belonging (Fields & Cunningham-Williams, 2021; LaPalme et al., 2022). Despite rejections and the barriers to success, Lyz continued trying to effect change.

More than just being resilient and focused, Lyz was a caring, funny friend. She nurtured and maintained relationships with those she cared about:

Lyz and I became friends when she reached out to me during a conference, an environment that I (and I think many others) find very uncomfortable. At this conference, Lyz was a keynote speaker and was surrounded by friends, yet she made the effort to connect, inviting me to spend time with her and her loved ones, creating comfort for me in an uncomfortable situation. That day, I became incredibly lucky to call Lyz my friend. From now on, in the spirit of Lyz’s kindness, I commit to connecting with and facilitating connections for folx who might be alone, lonely, or simply want to meet others in a safe, warm way, many of whom might identify with historically underrepresented communities. Lyz, thank you for creating such a safe space for me and for others. We will carry this on in your honor (N. Syed, personal communication, July 28, 2024).

## Lyz’s Legacy

As much as Lyz accomplished, she still had more to offer. She remained deeply reflective about the future of ABA and the need to welcome diverse forms of knowledge and experience. She also advocated for more compassionate, constructive conversations across our professional community. In the final chapter of her life, Lyz—like many in our field—grappled with the personal toll of persistent advocacy. By sharing her story, we honor not only what she gave to the field, but also what she still hoped to change.

Lyz’s legacy invites us to continue the work with honesty, humility, and care. She inspires us to be kind, prioritize relationships, and share perspectives. We honor and miss her compassion, humor, and humility. Even so, her work continues to impact the field, inspiring future generations to prioritize DEI. According to one student,

I saw Dr. Elizabeth [Hughes] Fong as a role model. This was due to her compassion and how she wanted positive changes in the field of ABA, while also prioritizing her responsibilities as a mother. I aspire to be like her and work hard to try to continue addressing the concerns she had about the field when I become a BCBA. She was not only brilliant, but she made live classes enjoyable due to her humor and personality. As a mother myself, I was deeply impressed with her ability to balance her many responsibilities and still make the time to attend her daughter’s matches at school. Her passing deeply affected me (M. Yashita, personal communication, May 1, 2024).

ABA practitioners continue to need help balancing their many varied responsibilities. They face burnout, compassion fatigue, and exhaustion, especially when engaged in DEI and social justice efforts. Self-care and wellness are not luxuries. They are critically important to maintaining health and long-term career satisfaction (e.g., Awa et al., 2010; Ko et al., 2023; Mohammed & Cummings, 2024). “It’s not like you attend a one-hour webinar. And now, yes, you changed, you’re woke. It’s continuing to work on yourself and self-awareness” (Hughes Fong, 2022). Lyz taught us that cultural sensitivity is a continuous and lifelong commitment. We hope this reflection and Lyz’s legacy inspire us to work toward a shared future that reflects her vision. Moving forward, we hope to engage openly with grief and healing, prioritize health and self-care, extend kindness and support to one another, and value interpersonal connection in our ongoing DEI efforts.

## Data Availability

This article does not describe research data.
